# Associations Between Dietary Patterns and Quality of Life in a Longitudinal Cohort of Colorectal Cancer Survivors

**DOI:** 10.3390/nu16223860

**Published:** 2024-11-12

**Authors:** Kristen S. Smith, Lisa M. Gudenkauf, Aasha I. Hoogland, Xiaoyin Li, Rachel Hoobler, Mary C. Playdon, Biljana Gigic, Brent J. Small, Brian D. Gonzalez, Laura B. Oswald, Doratha A. Byrd, K. Leigh Greathouse, Cornelia M. Ulrich, Christopher I. Li, David Shibata, Adetunji T. Toriola, Anita R. Peoples, Erin M. Siegel, Jane C. Figueiredo, Heather S. L. Jim, Sylvia L. Crowder

**Affiliations:** 1 Department of Health Outcomes and Behavior, Moffitt Cancer Center, Tampa, FL 33612, USAlisa.gudenkauf@moffitt.org (L.M.G.); aasha.hoogland@moffitt.org (A.I.H.); shelly.li@moffitt.org (X.L.); brian.gonzalez@moffitt.org (B.D.G.); laura.oswald@moffitt.org (L.B.O.); heather.jim@moffitt.org (H.S.L.J.); 2 Department of Nutrition and Integrative Physiology, College of Health, University of Utah, Salt Lake City, UT 84112, USA; rachel.hoobler@utah.edu (R.H.); mary.playdon@hci.utah.edu (M.C.P.); 3 Cancer Control and Population Sciences Program, Huntsman Cancer Institute, Salt Lake City, UT 84103, USA; 4 Department of General, Visceral and Transplantation Surgery, Heidelberg University Hospital, 69120 Heidelberg, Germany; biljana.gigic@med.uni-heidelberg.de; 5 School of Nursing, University of North Carolina at Chapel Hill, Chapel Hill, NC 27599, USA; brent.small@unc.edu; 6 Department of Cancer Epidemiology, H. Lee Moffitt Cancer Center and Research Institute, Tampa, FL 33612, USA; doratha.byrd@moffitt.org (D.A.B.); erin.siegel@nih.gov (E.M.S.); 7 Human Science and Design, Robbins College of Health and Human Sciences, Baylor University, Waco, TX 76798, USA; leigh_greathouse@baylor.edu; 8 Huntsman Cancer Institute, University of Utah, Salt Lake City, UT 84103, USAanita.peoples@hci.utah.edu (A.R.P.); 9 Department of Population Health Sciences, University of Utah, Salt Lake City, UT 84112, USA; 10 Division of Public Health Sciences, Fred Hutchinson Cancer Center, Seattle, WA 98109, USA; cili@fredhutch.org; 11 Department of Surgery, University of Tennessee Health Science Center, Memphis, TN 38163, USA; dshibata@uthsc.edu; 12 Department of Surgery, Washington University St. Louis, St. Louis, MO 63110, USA; a.toriola@wustl.edu; 13 Department of Medicine, Samuel Oschin Comprehensive Cancer Institute, Cedars Sinai Medical Center, Los Angeles, CA 90048, USA; jane.figueiredo@cshs.org

**Keywords:** cancer survivors, dietary patterns, quality of life, colorectal cancer

## Abstract

Purpose: To characterize dietary patterns and examine associations with cross-sectional and longitudinal changes in quality of life (QOL) over approximately one year after colorectal cancer (CRC) diagnosis. Methods: The ColoCare Study is an international, multi-center, prospective cohort study of newly diagnosed CRC survivors of any stage. A subset of participants with CRC in the United States completed patient-reported outcome measures at 6- and 12-months post-enrollment, including the Food Frequency Questionnaire (FFQ) and European Organization for the Research and Treatment of Cancer Quality of Life Questionnaire (EORTC QLQ-C30). Dietary patterns at 6 months (around the time of treatment completion) were identified using Principal Component Analysis (PCA) with varimax rotation. Adherence scores were calculated for participants within each dietary pattern, with higher scores indicating higher adherence. Mixed models were used to examine the effect of each dietary pattern on changes in QOL at 6- and 12-month follow-ups, controlling for cancer stage, biological sex, body mass index (BMI), smoking status, and age. Results: Participants (N = 174) were, on average, 56 ± 14 years old and were mostly female (51.5%), stage III or IV (51.7%), never smokers (60.2%), non-Hispanic (97.1%), and White (83.3%) with a BMI of 27.9 ± 6.1 kg/m^2^. PCA revealed two emerging dietary patterns: “Western diet”, characterized by processed meats, refined grains, and sugars, and “Prudent diet” characterized by lean proteins, fruits, and vegetables. Higher adherence to a Western diet was associated with worse social functioning at 6-month follow-up (FE = −12.6, *p* = 0.010). Loss of appetite from 6 to 12 months was associated with higher adherence to both the Western and Prudent dietary patterns (FE = 1.5, *p* = 0.044; FE = 1.3, *p* = 0.046, respectively). Neither dietary pattern was associated with global QOL score at 6- or 12-month follow-up (*p*’s > 0.05). Conclusions: Among CRC survivors in the United States, the Western diet was concurrently associated with worse social functioning. Loss of appetite was reported by CRC survivors following both dietary patterns, suggesting that loss of appetite may be a global experience for CRC survivors during this timeframe. Further research is needed to understand specific social challenges experienced by CRC survivors and develop supportive care interventions to address appetite and nutritional concerns.

## 1. Introduction

Globally, colorectal cancer (CRC) is the second leading cancer among women and third among men, with almost 2 million new cases in 2020 [1,2]. Improved access to guideline-concordant screening, diagnostic tests, and high quality treatment have decreased mortality rates in the last decade [3] and contributed to a growing population of CRC survivors, defined as an individual diagnosed with CRC from the point of diagnosis onward [4]. Health-related quality of life (QOL) is among the primary concerns of survivorship. QOL not only serves as an independent prognostic factor for CRC survival [5,6,7,8], but is also an important independent clinical outcome alongside tumor progression, recurrence, and survival [9]. The growing population of CRC survivors and the increasing emphasis on QOL underscores the importance of identifying modifiable risk factors and employing supportive care interventions to improve QOL and prolong survival. 

Diet quality is a modifiable lifestyle factor that has been positively associated with better QOL in CRC survivors [10,11]. Cumulative evidence among long-term survivors (i.e., ≥5 years) indicates that positive dietary changes can improve QOL, including physical and social functioning [12,13,14,15]. However, this research was limited to long-term survivorship and conducted in a heterogenous sample of cancer survivors across multiple cancer types. There is limited research investigating dietary patterns and QOL in short-term survivors (i.e., during the transition off treatment) with CRC. 

A recent systematic review showed that dietary information is a valued and desired component of survivorship care [16], and survivors express interest in receiving tailored dietary recommendations from their healthcare providers [17]. Indeed, the time of diagnosis could serve as a “teachable moment” and time to empower patients by offering recommendations for diet and other health-related behaviors [18]. Yet, CRC survivors participating in focus groups report a lack of information on dietary recommendations [19], and CRC survivors report inconsistent information, poor timing, insufficient or irrelevant recommendations, and/or difficult to understand material [17,20,21]. Limited provision of dietary recommendations is concerning, particularly given the role of diet in CRC and current dietary behaviors among CRC survivors. Estimates suggest that over 90% of CRC survivors do not meet recommendations to eat ≥5 servings/day of plant-based foods and limit consumption of red and processed meats to ≤3 servings/week [22]. 

To advance this field and inform evidence-based recommendations, researchers must elucidate specific dietary patterns among CRC survivors and determine how different dietary patterns relate to key survivorship outcomes, such as QOL [23]. Thus, this secondary analysis aimed to characterize dietary patterns among CRC survivors in the United States who were enrolled in a longitudinal cohort study and examine associations between diet quality and changes in QOL within approximately one year after diagnosis. We hypothesized that a healthier dietary pattern (e.g., high in fruits, vegetables, lean meats) would be associated better QOL, while an unhealthy dietary pattern (e.g., high in processed meats, refined grains, sugars) would be associated with worse QOL.

## 2. Methods

### 2.1. Study Sample

The study protocol was approved by Institutional Review Boards at all respective recruitment sites. Study participants were recruited as a part of the ColoCare Study cohort (NCT02328677) between December 2009 and August 2021, as close to diagnosis as possible. All participants provided written informed consent. Study design, eligibility criteria, and primary endpoints have been previously described [24,25,26,27]. In the current study, participants were selected from three study sites in the United States (i.e., Huntsman Cancer Institute (HCI) in Salt Lake City, UT, USA; Washington University School of Medicine (WUSM) in St. Louis, MO; and Cedars-Sinai Medical Center (CSMC) in Los Angeles, CA, USA). The subset of participants included in this secondary analysis completed a (1) Fred Hutch Food Frequency Questionnaire (FFQ) [28] at 6-month post-enrollment follow-up and (2) European Organization for the Research and Treatment of Cancer Quality of Life Questionnaire (EORTC QLQ-C30) [29] at 6- and 12-month follow-ups. 

### 2.2. Measures

#### 2.2.1. Demographic and Clinical Characteristics 

Questionnaires were used to collect participants’ self-reported demographic information at enrollment, including age at diagnosis; biological sex at birth; race; ethnicity; height and weight, used to calculate body mass index (BMI, kg/m^2^); and smoking status. Clinical characteristics (i.e., stage at diagnosis, primary tumor site, and adjuvant and neoadjuvant cancer treatments) were abstracted via medical record review.

#### 2.2.2. Quality of Life

QOL was assessed at 6 and 12 months post-enrollment using the (EORTC QLQ-C30) [29], designed to measure QOL in all cancer patients. The EORTC QLQ-C30 provides a global health status score, five functional scale scores (i.e., physical, role, emotional, cognitive, and social functioning), three symptom scale scores (i.e., fatigue, nausea and vomiting, and pain), and six item scores for self-reported symptoms (i.e., dyspnea, insomnia, appetite loss, constipation, diarrhea, and financial difficulties). Consistent with the scoring manual [30], scale scores were transformed to provide scores ranging from 0 to 100, with higher scores indicating greater severity of the construct being measured. Higher global health scores and functional scale scores indicate better QOL, whereas higher symptom scores indicate greater intensity of symptoms (and therefore worse QOL). 

#### 2.2.3. Dietary Intake

Dietary intake was assessed using the Food Frequency Questionnaire (FFQ) at six months post-enrollment. The FFQ is a patient-reported outcome measure developed by the Nutrition Assessment Shared Resource (NASR) of Fred Hutchinson Cancer Center and has been described elsewhere [31]. In short, the FFQ reflects eating frequency using predefined portion sizes for 125 food items over the past 12 months using a self-reported nine-point scale of “never”, “one per month”, “two to three per month”, “one per week”, “two per week”, “three to four per week”, “five to six per week”, “one per day”, and “two or more per day”. The Nutrient Data System for Research (NDSR), developed by the University of Minnesota’s Nutrition Coordinating Center [28], was used to calculate food nutrients. Daily intake of each food item in grams per day was calculated as the frequency of food item consumption multiplied by portion size. Consistent with other dietary pattern studies, food items were normalized for daily caloric intake, and adjusted to reflect a standard amount of calories to ensure the analysis did not bias by differing caloric intake levels [32].

### 2.3. Statistical Analysis 

Descriptive statistics, including means, standard deviations, frequencies, and percentages, were calculated for demographic and clinical variables. Principal Component Analysis (PCA) was used to derive dietary patterns explaining the maximum proportion of variance in the correlation matrix. An orthogonal transformation (Varimax) was conducted to rotate the correlation matrix and produce an efficient and interpretable loading structure. We determined the number of factors to retain by evaluating eigenvalues of the correlation matrix, scree plots, and intuitive interpretation of the factors. Factor scores for each identified pattern were calculated for all participants, with a higher factor score indicating greater tendency to adhere to a given dietary pattern. Food items with a factor loading ≥ 0.30 were considered to be a major contributor to the overall dietary pattern. Each dietary pattern was characterized across quartile of intake, with Q1 representing the lowest quartile and Q4 representing the highest quartile of intake. *T*-tests explored differences among dietary patterns and clinical and demographic characteristics. 

Linear random mixed-effects models were used to examine the association of dietary patterns with changes in QOL from six to 12 months (fit by quartile of exposure). Linear regression was used to calculate cross sectional associations between dietary patterns in quartiles (categorical variable) and QOL (continuous variable). Models were adjusted for theoretical confounders selected *a priori*, including cancer stage, biological sex, BMI (kg/m^2^), smoking status, and age at diagnosis. Statistical significance was set at *p <* 0.05. All analyses were conducted using SAS 9.4 (SAS Institute, Cary, NC, USA).

## 3. Results

A total of 174 participants met inclusion criteria for the present study. Participants were a mean age of 56 ± 14 years old and were mostly female (51.5%), non-Hispanic (97.1%), and White (83.3%) with an average BMI of 27.9 ± 6.1 kg/m^2^. Most participants were non-smokers (60.2%) or former smokers (32.8%), while 7% were current smokers. Demographic and clinical characteristics are shown in Table 1.

Food item factor loadings are reported in Supplementary Table S1. Two major dietary patterns were revealed via PCA: (1) a “Western diet” characterized by higher consumption of processed meats, refined grains, and sugars; and (2) a “Prudent diet” characterized by lean proteins, fruits, and vegetables. Dietary patterns (Western vs. Prudent) did not differ by ethnicity, site, stage, or BMI categories (*p*-values > 0.05). However, between-group differences in dietary pattern adherence by biological sex found that males were more likely to follow a Western diet than females (*p* = 0.001). 

**Figure 1:**
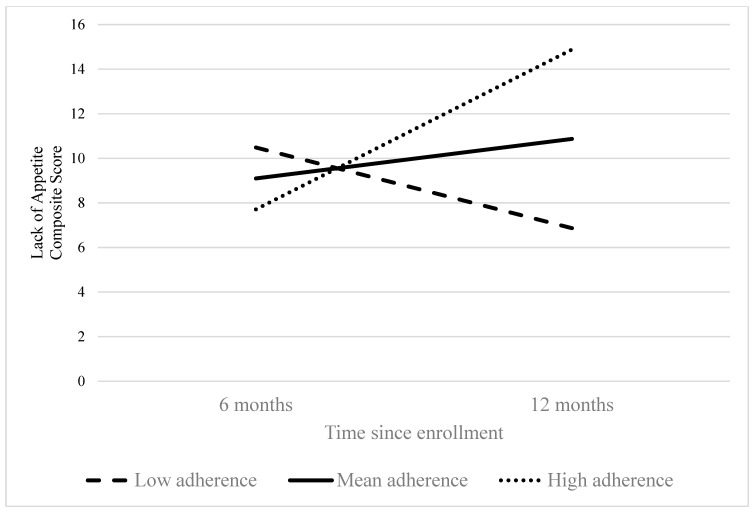
Changes in lack of appetite by level of adherence to a Western dietary pattern during transition off treatment in United States colorectal cancer survivors (*n* = 174).

**Figure 2:**
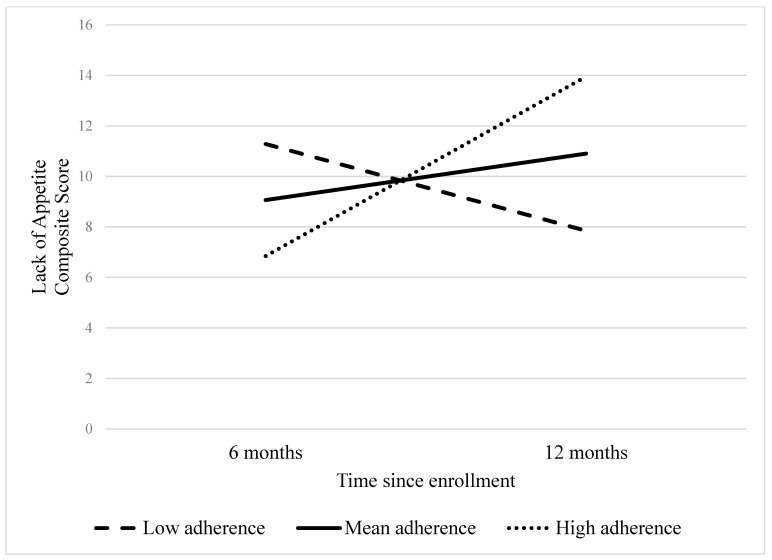
Changes in lack of appetite by level of adherence to a prudent dietary pattern during transition off treatment in United States colorectal cancer survivors (*n* = 174).

Associations between derived dietary patterns and EORTC QOL items are reported in Table 2. There were no significant relationships between dietary patterns and overall QOL. Higher adherence to a Western diet was associated with worse social functioning at six months (fixed effect (FE) = −12.6, *p* = 0.01). Greater loss of appetite from 6 to 12 months was associated with higher adherence to both Western and Prudent dietary patterns (FE = 1.5, *p* = 0.044; FE = 1.3, *p* = 0.046, respectively), Figure 1 and Figure 2. There were no other significant cross-sectional or longitudinal associations between QOL and dietary patterns (*p* values > 0.05). 

## 4. Discussion

To our knowledge, this is the first prospective cohort study to examine associations between diet quality and QOL in US CRC survivors transitioning off treatment. Herein, we identified two emerging dietary patterns: (1) Western diet, characterized by higher intake of processed meats, refined grains, and sugars (see Supplemental Table S1), and (2) Prudent diet, characterized by lean proteins, fruits, and vegetables (see Supplemental Table S1). Higher adherence to a Western dietary pattern was associated with less functioning in social roles at six months post-enrollment; it is possible this may be a spurious correlation and warrants further research. Surprisingly, higher adherence to both the Western and Prudent dietary patterns were associated with greater appetite loss. CRC survivors who experience appetite loss may be more willing to consume a variety of foods with different taste and textures in an effort to stimulate their appetite and make foods more appetizing. 

Notably, results of the present study differ somewhat from those of a German subsample from the same ColoCare cohort study [25]. While two dietary patterns were identified in our U.S. sample (Western diet and Prudent diet), four dietary patterns were identified in the German study: (1) Western diet, (2) fruit and vegetable (i.e., Prudent diet), (3) bread and butter diet, and (4) high-carbohydrate diet. Additionally, higher adherence to a Western diet was associated with loss of appetite in our U.S. subsample but associated with worsened physical functioning, constipation, and diarrhea in the German subsample [33]. Study differences may be attributable in part to cultural variances and the use of differing FFQ measures. Notably, social functioning variables were not explored in the German cohort; further investigation is needed to determine whether these patterns hold cross-culturally. 

The current study did not find a significant relationship between dietary patterns and overall QOL. This result contrasts with previous research demonstrating significant impacts of diet on QOL among CRC survivors [10,11,33,34]. Specifically, a U.K. study found that CRC survivors who consumed more fruits and vegetables (i.e., ≥5 servings/day) reported better QOL [35]. The lack of association between dietary pattern and overall QOL in the current study might be partially explained by social difficulties found among those adhering to a Western diet, as lower social functioning has been previously associated with greater risk for CRC and is a key factor that hinders engagement in health behaviors, like eating a healthy diet [36].

Following a Western diet during the transition off treatment was associated with greater loss of appetite. The previous literature has established a correlation between Western diet and appetite dysregulation [37,38,39]. Excessive intake of fat and sugar (consistent with a Western diet) can disrupt hippocampal function, leading to altered eating behaviors [37,38,39]. As overconsumption continues, hippocampal dysfunction worsens, further impacting appetite regulation [40,41]. Neurologic factors were not assessed in the present study; however, the association between Western diet and loss of appetite in CRC survivors contributes to the expanding body of literature linking the Western diet to poor appetite regulation [40,41].

Strengths of the present study include the prospective cohort design, inclusion of all CRC disease stages, and use of rigorous, validated patient-reported outcome measures of dietary intake and QOL. Several limitations must also be noted. As with many patient-reported outcome measures that require recall, recall bias and human error may have been introduced. While important associations between 6-month diet and QOL factors were identified in the present study, changes in diet quality over time could not be assessed given that diet data were not collected at 12 months post-enrollment. Lastly, the study design allowed for examination of important associations, but direction of causality could not be inferred. It is well recognized that the relationship between diet and QOL is both complex and bidirectional. More research is needed to examine longitudinal relationships between dietary intake and QOL and examine the directionality of the relationship between diet intake and QOL in order to establish evidence-based nutrition recommendations [42].

## 5. Conclusions

Herein, we characterized dietary patterns among United States CRC survivors and identified two distinct dietary patterns: Western diet and Prudent diet. Adherence to a Western dietary pattern was associated with greater social difficulties. Higher adherence to both Western and Prudent dietary patterns was associated with a greater loss of appetite during the transition off treatment. More research is needed to understand the directionality relationship between dietary intake and QOL, establish evidence-based nutrition recommendations, and develop efficacious nutritional interventions.

## Figures and Tables

**Table 1:** Demographic and clinical characteristics of United States colorectal cancer survivors enrolled in the current study (*n* = 174).

Variable	Mean	Standard Deviation
**Age (years)**	56.8	14.4
Age range	29–88	
Body mass index (BMI)	27.9	6.1
**Variable**	***n***	**%**
**BMI Category**		
Underweight (<18.5)	6	3.4
Normal (18.5–25)	50	28.8
Overweight (25–30)	73	41.9
Obese (>30)	45	25.9
**Sex**		
Female	89	51.2
Male	85	48.8
**Race**		
White	145	83.4
Black/African American	20	11.4
Native American	1	0.6
Asian	7	4.0
Other	1	0.6
**Ethnicity**		
Non-Hispanic	169	97.1
Hispanic	5	2.9
**Smoking status**		
Non-smoker	103	60.2
Former smoker	56	32.8
Current smoker	12	7.0
**Tumor Site**		
Colon	125	71.8
Rectal	49	28.2
**Stage at diagnosis**		
0-1	40	23.0
2	44	25.3
3	68	39.1
4	22	12.6
**Adjuvant therapy**		
Yes	80	45.9
No	88	50.6
Missing	6	3.5
**Neoadjuvant therapy**		
Yes	58	33.3
No	111	63.8
Missing	5	2.9
**Surgery**		
Yes	158	90.8
No	13	7.5
Missing	3	1.7

**Table 2:** Mixed-effect model associations of dietary patterns with changes in quality of life during transition off treatment in United States colorectal cancer survivors * (*n* = 174).

	6 Months				12 Months	
	Western		Prudent		Western	Prudent
EORTC QLQ-C30 Item	Estimate (SE)	*p*-Value	Estimate (SE)	*p*-Value	*p*-Value	*p*-Value
Global quality of life	0.84 (3.87)	0.830	2.62 (3.36)	0.440	0.827	0.280
**Functional Scales**						
Physical functioning	−0.56 (3.10)	0.859	2.89 (2.76)	0.302	0.295	0.610
Role functioning	−1.50 (4.91)	0.762	−1.54 (4.31)	0.723	0.164	0.795
Emotional functioning	−4.06 (3.36)	0.235	−0.88 (2.98)	0.770	0.833	0.895
Cognitive functioning	−5.54 (4.03)	0.177	−2.20 (3.53)	0.537	0.684	0.537
Social functioning	−12.55 (4.65)	0.010 ^#^	−2.54 (4.07)	0.537	0.228	0.908
**Symptom Scales/Items**						
Fatigue	−0.56 (4.25)	0.896	3.49 (3.72)	0.353	0.216	0.764
Nausea and vomiting	3.02 (2.92)	0.308	−0.12 (2.51)	0.961	0.609	0.116
Pain	3.29 (5.08)	0.521	−5.09 (4.41)	0.256	0.698	0.330
Dyspnea	6.19 (4.21)	0.149	4.08 (3.71)	0.279	0.469	0.236
Insomnia	−4.86 (5.44)	0.377	1.09 (4.68)	0.818	0.558	0.967
Appetite loss	−2.27 (4.28)	0.598	−3.31 (3.72)	0.378	0.044 ^#^	0.046 ^#^
Constipation	−4.87 (4.34)	0.269	5.24 (3.75)	0.170	0.930	0.479
Diarrhea	6.11 (4.26)	0.160	2.07 (3.71)	0.580	0.114	0.818
Financial difficulties	8.90 (4.73)	0.068	−1.89 (4.15)	0.651	0.479	0.192

* controlling for cancer stage, biological sex, body mass index (kg/m^2^), smoking status, and age at diagnosis. ^#^ indicates statistical significance.

## Data Availability

Data are available upon reasonable request from the corresponding author. The data are not publicly available due to participant privacy concerns and institutional policies.

## Supplementary Materials

The following supporting information can be downloaded at: https://www.mdpi.com/article/10.3390/nu16223860/s1, Table S1: Dietary pattern factor loading matrix; Figure S1: Scree plot of eigenvalues resulting from principal component analysis.
