# Supplementary material for: Associations Between Dietary Patterns and Quality of Life in a Longitudinal Cohort of Colorectal Cancer Survivors
Source: Nutrients. 2024 Nov 12;16(22):3860. doi: 10.3390/nu16223860 (PMC11597155; doi:10.3390/nu16223860)
Supplement: Supplementary file 1 [file nutrients-16-03860-s001.zip › nutrients-3298659-Figure S1.pdf]

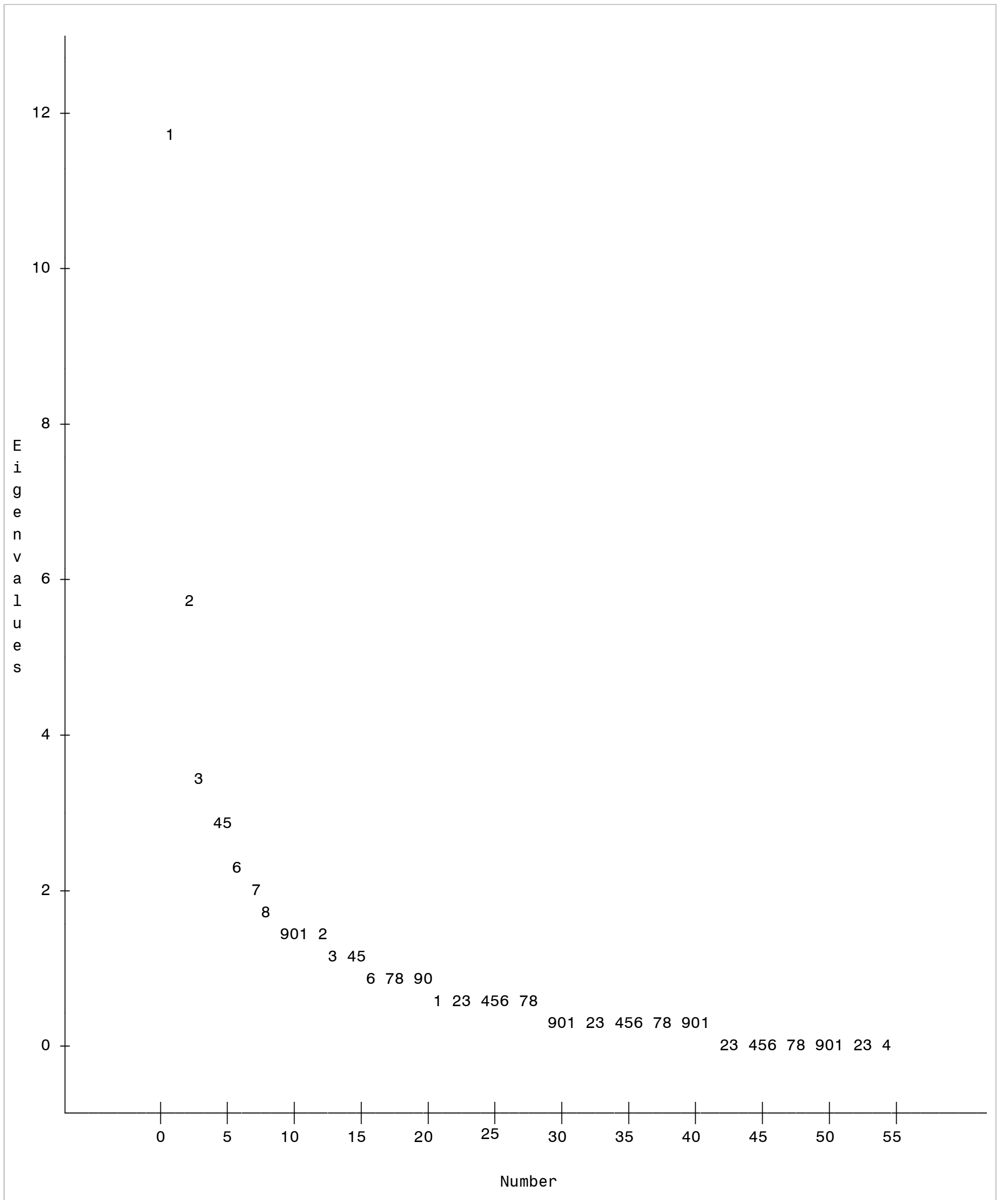

Supplemental Figure S1. Scree plot of eigenvalues resulting from principal component analysis
