# Supplementary material for: Associations Between Dietary Patterns and Quality of Life in a Longitudinal Cohort of Colorectal Cancer Survivors
Source: Nutrients. 2024 Nov 12;16(22):3860. doi: 10.3390/nu16223860 (PMC11597155; doi:10.3390/nu16223860)
Supplement: Supplementary file 1 [file nutrients-16-03860-s001.zip › nutrients-3298659-Table S1.pdf]

Supplementary Table S1. Dietary pattern factor loading matrix

| Food Item | Western | Prudent |
|---|---|---|
| Refined Grains, dry flour | 0.764* | 0.279 |
| Fried Chicken | 0.762* | -0.052 |
| Refined Pasta | 0.760* | 0.186 |
| Fried Potatoes | 0.725* | 0.053 |
| Fried Fish | 0.719* | -0.022 |
| Lean Beef | 0.715* | -0.045 |
| Pizza | 0.710* | 0.225 |
| Honey, jam, jelly | 0.686* | 0.203 |
| Beef | 0.680* | 0.033 |
| Other Bread, Refined | 0.665* | 0.076 |
| Reduced Cheese | 0.659* | 0.240 |
| Reduced Sauce, Condiments | 0.652* | 0.032 |
| Regular Margarine | 0.614* | 0.104 |
| Cold cuts, Sausage | 0.590* | -0.205 |
| Refined Dessert | 0.576* | 0.038 |
| Regular Gravy | 0.574* | 0.020 |
| Lean Cold cuts, Sausage | 0.503* | -0.170 |
| Organ Meat | 0.499* | 0.024 |
| Whole Cheese | 0.483* | 0.144 |
| Regular Cured pork | 0.436* | -0.213 |
| Sweet Fruit Drink | 0.418* | 0.012 |
| White Potato | 0.401* | 0.230 |
| Vegetable Snack | 0.378* | 0.075 |
| Refined Loaf Bread | 0.377* | 0.033 |
| Eggs | 0.376* | -0.152 |
| Cream Soup | 0.366* | 0.131 |
| Ready-to-eat Refined Sweet Cereal | 0.341* | -0.012 |
| Sugar | 0.330* | 0.216 |
| Citrus Juice | 0.300* | 0.235 |
| Other Vegetables | 0.180 | 0.721* |
| Chocolate Candy | 0.154 | 0.329* |
| Citrus Fruit | 0.104 | 0.667* |
| Other Fruit | 0.034 | 0.731* |
| Unsweet Coffee | 0.025 | 0.318* |
| Nut seed Butter | -0.072 | 0.617* |
| Deep Yellow Vegetables | -0.101 | 0.684* |
| High-fat Fruit | -0.109 | 0.462* |
| Meat Alternative | -0.114 | 0.628* |
| Dark Green Vegetables | -0.132 | 0.790* |
| Low-fat Sweet Yogurt | -0.132 | 0.531* |
| Ready-to-eat Whole Grain Cereal | -0.149 | 0.418* |
| Legumes | 0.438* | 0.553* |
| Tomatoes | 0.408* | 0.524* |
| Salad Dressing | 0.366* | 0.470* |
| Fish | 0.320* | 0.450* |
| Whole grain bread | 0.344* | 0.435* |

*Factor loading > 0.30 considered to be a major contributor to the overall pattern.
